# Identifying the window of aggressive postpartum breast cancer based on the 21-gene Oncotype DX® test in women with HR+, HER2-negative breast cancer

**DOI:** 10.1038/s41523-026-01002-2

**Published:** 2026-07-01

**Authors:** Shiliang Zhang, Katelyn J. Queen, Niharika Duggirala, Eric Yang, Marla Lipsyc-Sharf, Jennifer Racz, John Bennett, Kaden Hong, Yashila Suresh, Charlotte Hamilton, Meghana Pitta, Nicole Chien, Emily La, Christy A. Russell, Aditya Bardia, Nimmi S. Kapoor

**Affiliations:** 1 Division of Hematology-Oncology, Department of Medicine, University of California, Los Angeles, Los Angeles, CA USA; 2 David Geffen School of Medicine, University of California, Los Angeles, Los Angeles, CA USA; 3 College of Letters and Science, University of California, Los Angeles, Los Angeles, CA USA; 4 Medical Affairs, Abbott, Redwood City, CA USA; 5 Clinical Biostatistics, Abbott, Redwood City, CA USA; 6 Department of Surgery, University of California, Los Angeles, Los Angeles, CA USA

**Keywords:** Cancer, Oncology

## Abstract

Postpartum breast cancer (PPBC) is associated with worse outcomes in young women with breast cancer and demonstrates distinct transcriptomic features, yet a temporal definition based on gene expression has not been established. We conducted a single-institution retrospective cohort study to evaluate the impact of postpartum status on the 21-gene Oncotype DX Recurrence Score® (RS) in hormone receptor-positive, HER2-negative young women with breast cancer. Among 385 patients, 153 were nulliparous, 62 were more than 10 years postpartum, and 170 were within 10 years postpartum. Diagnosis within one year of childbirth was associated with a 13.2-point higher RS compared with nulliparous patients (*p* < 0.001). Defining PPBC as within 3 years postpartum was associated with increased odds of RS category shift (OR 2.83, *p* = 0.001). In the context of contemporary adjuvant treatment, with patients diagnosed within three years postpartum more likely to receive chemotherapy, ovarian function suppression, and CDK4/6 inhibitors, differences in IDFS associated with time since last childbirth did not result in significantly worse IDFS within the follow-up period (89.0% in patients with PPBC up to 3 years vs. 93.7% in all other patients *p* = 0.39). These findings indicate that tumors diagnosed within the first year postpartum have significantly elevated RS, and that the period of most aggressive PPBC, as reflected by gene expression profiles, may arise in the more recently postpartum period, in the first 0–3 years postpartum.

## Introduction

In the United States, the incidence of breast cancer is rising, but the incidence among women <50 years is rising even faster, a trend that may in part be driven by the rising age at first childbirth^1,2^. Pregnancy exerts a dual effect on breast cancer incidence - risk transiently increases during the immediate postpartum period before eventually declining, and in women who complete their first birth after age 35, breast cancer risk remains elevated above that of nulliparous women^3^. Not only does incidence of breast cancer increase in the postpartum period, breast cancers diagnosed during the postpartum period are also more likely to exhibit aggressive clinical and pathologic features and worse outcomes for distant disease recurrence and overall survival^4–7^.

Research on PPBC and pregnancy-associated breast cancer (PABC) is limited by inconsistent definitions, resulting in heterogeneous cohorts. For example, PABC generally includes breast cancers diagnosed during pregnancy as well as those occurring in the early postpartum period, typically within 1–2 years after delivery. More recently, however, there has been a push to refine the definition of PABC by distinguishing gestational breast cancers—also called pregnancy-related breast cancers (PrBC)—from breast cancers diagnosed in the postpartum setting (PPBC)^8^. This distinction is supported by cohort studies showing that PPBC is associated with worse outcomes compared to PrBC^9^. Retrospective studies further indicate that cancers diagnosed up to 5 years postpartum are linked to lower overall survival and higher risk of distant recurrence, supporting an extended definition of PPBC to include this timeframe. Some studies have even evaluated PPBC up to 10 years postpartum, demonstrating persistent associations with adverse outcomes, including reduced metastasis-free survival and more aggressive tumor features^10–13^. These data have prompted efforts to characterize the distinct postpartum biology underlying the increased risk of aggressive disease in PPBC.

Recent studies comparing the transcriptomes of PPBC patients with those of nulliparous women, as well as PPBC versus pregnancy-related breast cancer (PrBC) patients, have demonstrated that PPBC exhibits a distinct transcriptomic profile characterized by increased immune infiltration and increased cell-cycle gene expression^14,15^. In a small cohort comparing PPBC to nulliparous breast cancer patients, researchers found that even among HR+, HER2-negative tumors, PPBC exhibited higher-risk features, including reduced ER signaling^15^. In the same study, they found that a pseudo-21-gene score in PPBC patients (*n* = 9) was higher than in nulliparous breast cancer patients (*n* = 7, *p* = 0.034)^15^. The FLEX sub-study of HR+, HER2-negative PPBC, showed that patients diagnosed within 5 years postpartum were more likely to have MammaPrint High 2 tumors and less likely to have MammaPrint Low-risk tumors compared with nulliparous women or those diagnosed 6–10 years postpartum^16,17^. MammaPrint High 2 tumors are characterized by more aggressive biology and greater benefit from chemotherapy, suggesting that PPBC diagnosed within 5 years of childbirth may represent a biologically more aggressive subset of disease^17^. These results suggest that the distinct biological features of PPBC may be detected by validated commercial gene expression assays which could help guide clinical management in this higher-risk population.

In the present study, we aimed to build on prior work that evaluated a pseudo–21-gene result by using the commercially available Oncotype DX Breast Recurrence Score^®^ result—a commonly used gene expression assay in breast cancer for assessing recurrence risk and prediction of chemotherapy benefit in HR+, HER2-negative disease—to determine whether the score is elevated in postpartum breast cancer patients^18^. Specifically, we aimed to refine the temporal definition of PPBC by assessing whether Recurrence Score^®^ (RS) result elevation is concentrated within specific time intervals of postpartum breast cancer and whether this correlates with decreased disease-free survival. We also evaluated associated molecular and pathological features to identify biologically distinct subsets that may warrant tailored clinical management.

## Results

### Recurrence score result analyzed by parity status

The final analysis set included 385 women with HR+, HER2-negative breast cancer diagnosed at a single institution between July 1, 2015, and October 9, 2024. The mean age at diagnosis was 40.7 years (range: 26.6–45.9 years). Of these, 153 were nulliparous. Parous patients were categorized according to the interval between their most recent childbirth and breast cancer diagnosis: 85 were ≤5 years postpartum, 85 were >5 to ≤10 years postpartum, and 62 were >10 years postpartum. The recurrence score result was initially compared across these four groups. Clinical, demographic, and treatment characteristics stratified by parity status are summarized in Table 1.

**Table 1:** Characteristics of the patient cohort

	Nulliparous (*n* = 153)	≤5 years postpartum (*n* = 85)	>5 to ≤10 years postpartum (*n* = 85)	>10 years postpartum (*n* = 62)	*p*-value	Total (*n* = 385)
Age at diagnosis	39.2 [26.6, 45.9]	40.3 [29.9, 45.9]	42.4 [32.7, 45.8]	43.1 [36.1, 45.8]	<0.001***	40.7 [26.6, 45.9]
Age at first childbirth	NA	34.3 [17.0, 42.9]	30.9 [14.7, 40.1]	25.1 [15.4, 34.3]	<0.001***	30.6 [14.7, 42.9]
Grade						
1	29 (19.0%)	12 (14.1%)	23 (27.1%)	14 (22.6%)	0.261	78 (20.3%)
2	95 (62.1%)	48 (56.5%)	44 (51.8%)	34 (54.8%)		221 (57.4%)
3	29 (19.0%)	25 (29.4%)	18 (21.2%)	14 (22.6%)		86 (22.3%)
Stage					0.401	
1	74 (48.4%)	41 (48.2%)	50 (58.8%)	27 (43.5%)		192 (49.9%)
2	74 (48.4%)	39 (45.9%)	32 (37.6%)	30 (48.4%)		175 (45.5%)
3	5 (3.3%)	5 (5.9%)	3 (3.5%)	5 (8.1%)		18 (4.7%)
Ki67 category					0.010**	
0–19%	82 (53.6%)	38 (44.7%)	58 (68.2%)	36 (58.1%)		214 (55.6%)
≥20%	70 (45.8%)	47 (55.3%)	25 (29.4%)	25 (40.3%)		167 (43.4%)
Missing	1 (0.7%)	0 (0%)	2 (2.4%)	1 (1.6%)		4 (1.0%)
Received chemotherapy					0.003**	
Yes	45 (29.4%)	39 (45.9%)	17 (20.0%)	20 (32.3%)		121 (31.4%)
Missing	1 (0.7%)	0 (0%)	0 (0%)	1 (1.6%)		2 (0.5%)
Endocrine therapy					0.802	
Yes	137 (89.5%)	79 (92.9%)	80 (94.1%)	55 (88.7%)		351 (91.2%)
Missing	3 (2.0%)	0 (0%)	0 (0%)	1 (1.6%)		4 (1.0%)
CDK4/6 Inhibitors					0.590	
Yes	14 (9.2%)	12 (14.1%)	8 (9.4%)	5 (8.1%)		39 (10.1%)
Missing	1 (0.7%)	0 (0%)	0 (0%)	1 (1.6%)		2 (0.5%)
Ovarian suppression					0.073	
Yes	65 (42.5%)	49 (57.6%)	34 (40.0%)	24 (38.7%)		172 (44.7%)
Missing	5 (3.3%)	0 (0%)	0 (0%)	2 (3.2%)		7 (1.8%)
Follow-up time (months)	49.5 [2.0, 120.3]	50.1 [8.5, 113.9]	47.8 [8.7, 117.7]	41.9 [9.6, 109.1]	0.369	48.6 [2.0, 120.3]
Locoregional recurrence or second primary	6 (3.9%)	4 (4.7%)	4 (4.7%)	2 (3.2%)	0.967	16 (4.2%)
Distant recurrence	4 (2.6%)	4 (4.7%)	1 (1.2%)	4 (6.5%)	0.278	13 (3.4%)

Continuous variables summarized by mean [minimum, maximum]. Categorical variables summarized by count (percent). Increasing number of * represent statistical significance at alpha = 0.05, 0.01, 0.001; *p*-values from ANOVA if variable is continuous, chi-squared test if variable is categorical.

**Fig. 1:**
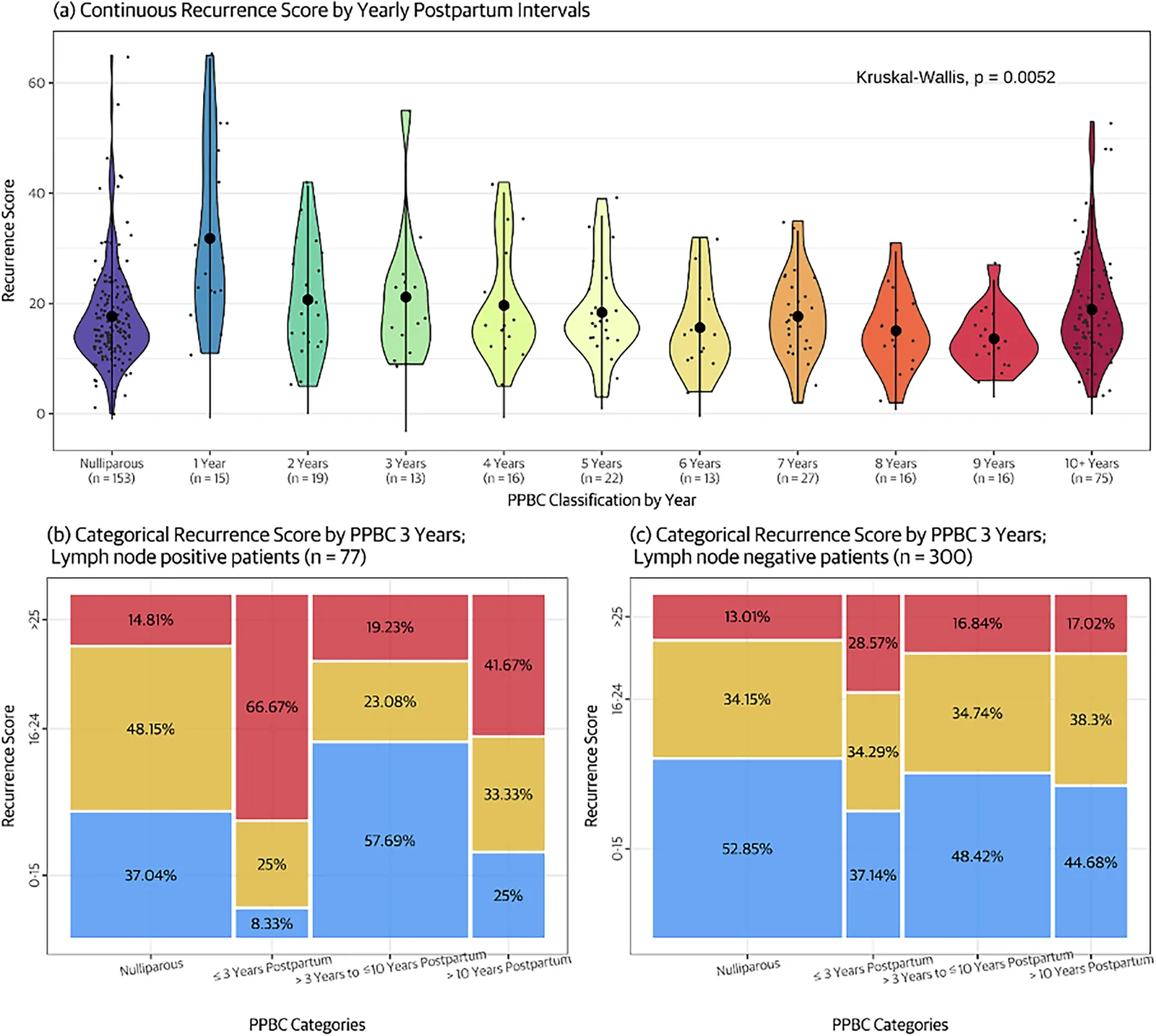
Recurrence score result and postpartum intervals. **a** Violin plot for recurrence score by PPBC classification. Each dot represents values for one patient. Kruskal-Wallis was used to test for global differences. **b** Mosaic plot for categorical recurrence score by postpartum category in lymph node positive patients. Values represent percentage of patients. **c** Mosaic plot for categorical recurrence score by postpartum category in lymph node negative patients.

Patients with PPBC diagnosed ≤5 years postpartum had a higher average Recurrence Score result by 4.38 points (95% CI: 1.84–6.92, *p* < 0.001) compared with nulliparous patients, while those with PPBC diagnosed >5 to ≤10 years and >10 years had an average increase of 0.66 points (95% CI: –1.97–3.30), and 2.70 points (95% CI: –0.30–5.71), respectively, neither of which were statistically significant. When the recurrence score result was further stratified by postpartum year, patients diagnosed in the first year postpartum had a significantly higher mean RS (13.08 points, 95% CI: 8.15–18.01) than nulliparous women (*p* < 0.001). In the second year postpartum, the mean recurrence score result remained elevated by 2.6 points compared with nulliparous patients (*p* = 0.25). A numerical increase of 4.5 points on average was also observed at 3 years postpartum, although these differences did not reach statistical significance (*p* = 0.10) (Fig. 1a, violin plot). Patients diagnosed in the first year postpartum also had significantly reduced ER and PR gene scores compared with nulliparous patients (*p* = 0.003 and *p* < 0.001, respectively). Notably, decreases in the ER and PR gene scores were also observed in the > 10 years postpartum group (Supplementary Figs. 2 and 3). The ER and PR gene scores correlated with ER and PR staining determined by IHC noted in surgical pathology reports (Pearson’s *R* = 0.51, 0.70, respectively; both *p* < 0.001).

Given the increase in Recurrence Score results observed up to 3 years postpartum, we re-categorized the PPBC cohort as ≤3 years postpartum (*n* = 47) and >3 to ≤10 years postpartum (*n* = 123). Redefining PPBC as ≤3 years postpartum increased the effect size to a mean Recurrence Score difference of 6.44 points compared with nulliparous patients (*p* < 0.001) (Table 2). The odds of being classified in a higher Recurrence Score category were significantly greater for patients with PPBC diagnosed ≤5 years postpartum compared with nulliparous patients (OR = 2.23, *p* = 0.002). When redefining PPBC as ≤3 years postpartum, the odds of a higher recurrence score category was increased (OR = 2.83, *p* = 0.001). An elevated odds of higher recurrence score category was also observed in patients diagnosed >10 years postpartum (*p* = 0.02). All associations were observed after controlling for age at diagnosis and lymph node status.

**Table 2:** Comparison of effect of different definitions of postpartum cohort on the recurrence score and invasive disease recurrence

	Continuous recurrence score		Categorical recurrence score		Invasive disease recurrence	
	[0.0, 65.0]		(0–15, 16–25, >25)			
PPBC definition	Beta (95% CI)	*p*-value	OR (95% CI)	*p*-value	HR (95% CI)	*p*-value
PPBC, ≤5 years pp						
≤5 years pp	4.38 (1.84, 6.92)	<0.001***	2.23 (1.33, 3.74)	0.002**	2.29 (0.86, 6.05)	0.10
>5 to ≤10 years pp	0.66 (−1.97, 3.30)	0.62	1.14 (0.66, 1.97)	0.63	2.30 (0.69, 7.66)	0.17
>10 years pp	2.70 (-0.30, 5.71)	0.078	2.04 (1.11, 3.75)	0.022*	4.38 (1.24, 15.51)	0.022*
PPBC, ≤3 years pp						
≤3 years pp	6.44 (3.37, 9.52)	<0.001***	2.83 (01.51, 5.34)	0.001**	2.19 (0.73, 6.58)	0.16
>3 to ≤10 years pp	0.95 (−1.39, 3.29)	0.42	1.28 (0.79, 2.07)	0.32	2.39 (0.82, 6.94)	0.11
>10 years pp	2.61 (−0.38, 5.59)	0.087	2.02 (1.10, 3.72)	0.023*	4.45 (1.24, 15.90)	0.022*

Models for recurrence score adjusted for pN stage (0–3) and age at diagnosis; Invasive disease-free survival model is adjusted for stage (1, 2+), age at diagnosis, and year of breast cancer diagnosis (2015–2020, 2021–2024); Reference group is nulliparous; Increasing number of * represent statistical significance at alpha = 0.05, 0.01, and 0.001.

*PPBC* postpartum breast cancer, *pp* postpartum, *OR* odds ratio, *CI* confidence interval, *HR* hazards ratio.

Among lymph node-positive patients (*n* = 77), the odds of moving into a higher Recurrence Score risk category were 8.30 times for those who had PPBC ≤ 3 years postpartum compared with nulliparous patients (*p* = 0.003, Fig. 1b). Among lymph node-negative patients (*n* = 300), the corresponding increase in odds was 2.21 when comparing patients with PPBC ≤ 3 years postpartum with nulliparous patients (*p* = 0.028, Fig. 1c). Risk categories were considered 0–15 for low, 16–25 for intermediate, and >25 for high risk.

### Postpartum year and tumor features

We evaluated additional tumor characteristics, including tumor stage, lymph node status, and Ki-67 expression (<20% vs. ≥ 20%), across cohorts defined by parity (nulliparous, PPBC ≤ 3 years, PPBC > 3 to ≤10 years, and PPBC > 10 years). Patients in the PPBC ≤ 3 years cohort had significantly higher odds of presenting with a higher pathologic grade compared with nulliparous patients (OR = 2.35, *p* = 0.009). No significant differences in odds of higher pathologic grade were observed in the PPBC > 3 to ≤10 years or >10-year cohorts compared to nulliparous patients. Similarly, no significant differences were identified across cohorts for tumor stage, lymph node status, or Ki-67 expression.

### Systemic therapy patterns by cohort

**Fig. 2:**
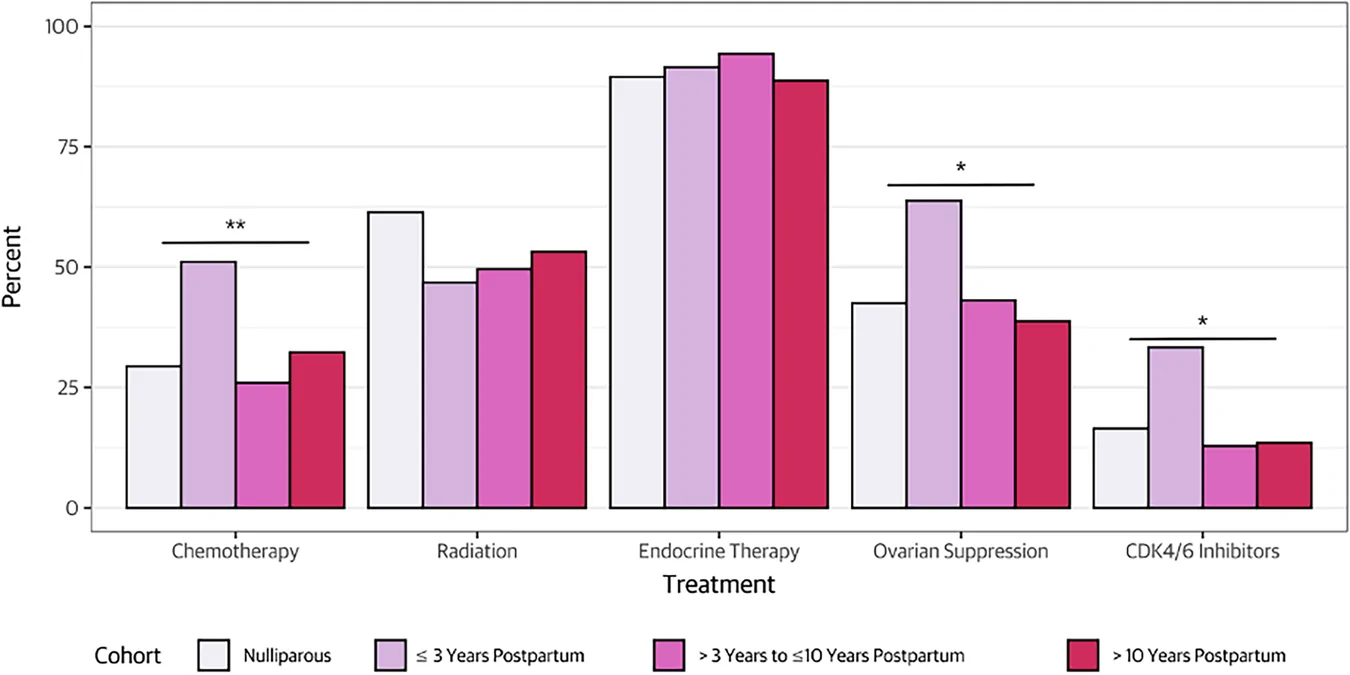
Prevalence of different systemic therapy among cohorts. Bar graph for various therapies based on postpartum category. Only patients diagnosed after the first CDK4/6 inhibitors were approved for initial treatment in early-disease, premenopausal women on October 12, 2021 were included for the CDK4/6 portion of the graph^25^. Horizontal bars represent significant differences in distribution of treatment based on PPBC category, using a chi-square test; * indicates *p* < 0.10 and ** indicates *p* < 0.05.

In our cohort, patients with PPBC ≤ 3 years postpartum demonstrated higher rates of aggressive systemic therapy, with a trend toward increased use of chemotherapy, ovarian function suppression, and CDK4/6 inhibitors, although these differences did not reach statistical significance (Fig. 2). Lymph node–positive patients received chemotherapy 62.3% of the time. Among lymph node–positive patients with low-risk RS disease, 50.9% received chemotherapy, compared with 90.9% of those with high-risk disease defined by RS > 25 (*p* = 0.001).

### Recurrence and survival

**Fig. 3:**
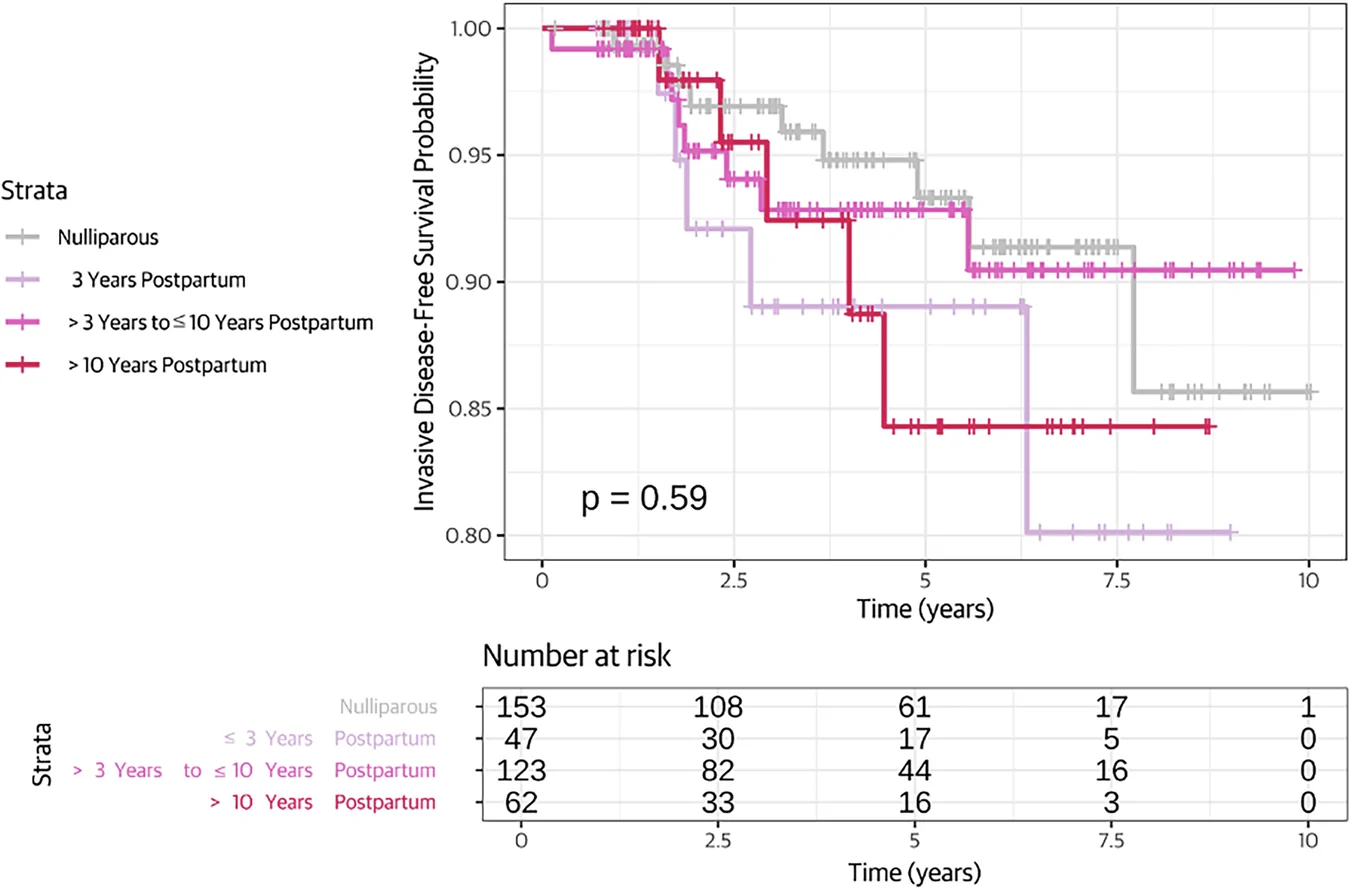
Invasive disease-free survival by cohort. Kaplan–Meier curve and risk table for invasive disease-free survival by PPBC classification. Patients were censored (vertical bars) at last follow-up. *P*-value calculated using a log-rank test for differences between groups.

The median follow-up time was 48.6 months (range: 2.1–122 months). During this period, there were 3 s primary breast malignancies, 13 local recurrence events and 13 distant recurrence events, occurring in a total of 27 unique patients. One death was recorded. Kaplan–Meier analysis demonstrated no significant differences in 4-year IDFS in the ≤3-year postpartum cohort compared with all other patients (HR 1.42, 95% CI: 0.53–3.77, *p* = 0.48). Hazard ratios for recurrence for PPBC categories compared to nulliparous patients can be seen in Table 2 and Fig. 3. A sensitivity analysis using recurrence-free survival (RFS), restricted to local recurrence, distant recurrence, and death and excluding second primary breast malignancies, yielded results consistent in direction and magnitude with the primary IDFS analysis (Supplementary Table 1 and Supplementary Fig. 5).

## Discussion

Current definitions of PPBC are based on meta-analyses and observational cohort studies that generally identify worse outcomes within predefined intervals, most commonly 0–5 years or up to 10 years postpartum. By conducting a year-by-year analysis and using a gene expression panel as a surrogate of tumor biology, we observed a significant increase in Recurrence Score results in patients diagnosed in the first year postpartum, as well as a non–statistically significant increase toward higher Recurrence Score results extending up to 3 years postpartum. In the context of contemporary adjuvant treatment, however, these molecular differences did not translate into significantly worse IDFS within the follow-up period in the PPBC cohort compared to nulliparous patients (4-year IDFS 89.0% vs. 93.7%, HR = 2.19, *p* = 0.16). Together, these data suggest that the window of greatest molecular risk may be more narrowly defined as 0–3 years postpartum, rather than the broader 0–5-year or expansive 0–10-year interval. The association of risk (as measured by RS) remains significant after adjusting for age and lymph node status, despite no differences in tumor or nodal status across cohorts. This suggests that, apart from tumor grade, traditional clinicopathologic features may not fully capture risk in this population, and that the recurrence score result provides additional prognostic value.

Our findings align with prior evidence from gene expression panels demonstrating greater recurrence risk in patients diagnosed closer to childbirth. For example, a recent Dutch study reported worse outcomes among patients diagnosed within 6 months of delivery compared with those diagnosed between 6 months and 1-year postpartum, while a Stanford study found poorer outcomes in patients diagnosed within the first 2 years postpartum compared with those diagnosed between 2 and 5 years^7,19^. Moreover, patients diagnosed while breastfeeding were observed to have inferior outcomes and more adverse clinicopathologic features. In our cohort, 15 patients were diagnosed with PPBC within the first year; breastfeeding data were available for nine of these patients, six of whom were not breastfeeding at diagnosis. These findings underscore the need to further evaluate differences between breast cancers diagnosed in the early postpartum period, during lactation, and after weaning, as well as in later postpartum intervals. The prevailing explanation for the aggressive biology of PPBC has centered on mammary gland involution—the process by which the lactating breast returns to a pre-pregnancy state. Involution is characterized by activation of wound-healing and inflammatory pathways that may promote tumor progression, angiogenesis, and lymph node metastasis, as well as the development of an immunosuppressive microenvironment^3,20^. However, the involution hypothesis alone may not fully account for the elevated risk observed among patients diagnosed in the first year postpartum. Many breast cancer diagnoses in the postpartum period occur after patients palpate a mass during lactation, highlighting the need for further investigation into how lactation status and duration influence tumor biology and outcomes. Increasing evidence suggests that lactation itself, and particularly its duration, plays a critical role in shaping mammary immune remodeling and subsequent cancer behavior. Recent data demonstrate that completion of a full reproductive cycle—including lactation and involution—induces long-term accumulation of tissue-resident memory CD8⁺ T cells in the breast, enhancing local immune surveillance. Consistent with this, longer total breastfeeding duration has been associated with greater tumor-infiltrating lymphocytes (TILs), improved survival in parous women with triple-negative breast cancer, and potentially enhanced response to immunotherapy. These findings highlight that reproductive immune remodeling extends beyond involution alone and underscore the need to delineate how lactation status and duration influence tumor biology across breast cancer subtypes^21^.

Overall, our findings underscore the need for more refined biological definitions of PPBC that incorporate molecular signatures in addition to outcomes-based intervals. A deeper understanding of gene expression patterns in young women is particularly important. Although the Recurrence Score result has been validated in both pre- and postmenopausal women, postmenopausal women represented the majority of participants in these trials. In TAILORx, only 4.6% of participants were under the age of 40, and approximately 27% were between 40 and 50 years of age^22^. In RxPONDER, approximately one third of patients were premenopausal^23^. Among premenopausal node-positive patients, interpretation of RS remains uncertain—all demonstrated a chemotherapy benefit, but it remains unresolved whether this benefit stems primarily from the cytotoxic effect of chemotherapy or from treatment-induced ovarian suppression. Ongoing trials such as OFSET (NCT05879926) aim to address this question. Importantly, our data show that a substantial proportion of premenopausal, node-positive patients in routine practice do not receive chemotherapy, indicating real-world variation in decision-making. Because PPBC is associated with higher Recurrence Score results in both node-negative and node-positive patients, determining whether the absence of PPBC identifies a subset of premenopausal women can safely omit chemotherapy is an important question to evaluate^18,24^.

Our results also highlight the importance of more comprehensive consideration of reproductive history in clinical practice. While age at menarche and age at first childbirth are routinely documented and associated with overall breast cancer risk, our analysis found no association between either factor and Recurrence Score results (Supplementary Fig. 4). This suggests that although these reproductive factors may influence incidence of breast cancer diagnosis, they may not increase the likelihood of biologically aggressive disease. By contrast, age at last childbirth may represent a critical clinical variable, as the interval since last childbirth appears to be associated with higher Recurrence Score results.

**Fig. 4:**
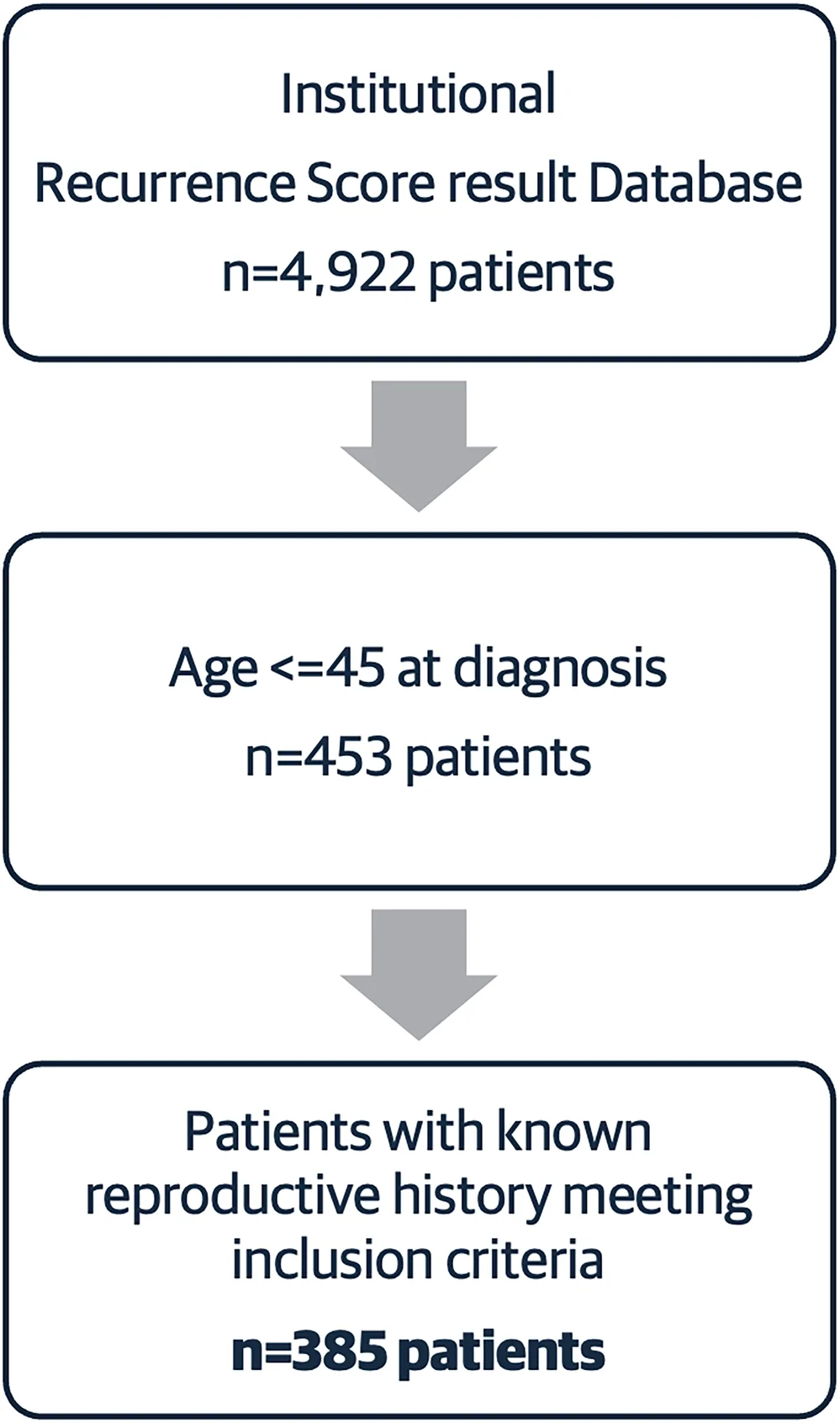
Consort-style flow diagram of patient selection.

Our study has several notable strengths, including the use of a large, well-annotated institutional dataset with detailed reproductive history and access to gene expression profiling through Oncotype DX^®^ testing. The year-by-year analysis of postpartum timing further allowed for a more granular assessment of how Recurrence Score result differs across the postpartum period. Our study also has several limitations. Our cohort is limited to patients who underwent RS testing, which may underrepresent those with higher-risk disease typically associated with larger tumors or greater nodal involvement—features more common in PPBC—as well as those with very low-risk disease for whom testing was deemed unnecessary. Accordingly, our study population likely reflects an intermediate risk subset of young women with breast cancer treated at a single institution. Additionally, parity data were obtained through retrospective review of clinical documentation, and 68 age-eligible patients (15%) were excluded due to incomplete reproductive history records (Fig. 4). Incomplete documentation most likely reflects limited longitudinal follow-up at our institution rather than any systematic relationship to disease or postpartum characteristics. The challenges encountered in obtaining parity data highlight the absence of standardized reporting of reproductive history including parity status and time since last childbirth, in both routine clinical documentation and prospective breast cancer trials, representing a critical gap that limits the field’s ability to study and characterize PPBC at scale. Finally, outcome analyses are limited by the small number of recurrence events (*n* = 29) and the relatively short follow-up period. This is particularly relevant for patients with HR+, HER2-negative disease, which is characterized by the potential for late recurrence extending over decades. In this context, the statistically significant increase in invasive disease recurrence hazard observed in patients diagnosed more than 10 years postpartum (HR = 4.45, 95% CI: 1.24–15.90, *p* = 0.022) warrants cautious interpretation. The wide confidence intervals and small number of events in this subgroup suggest potential model instability. Notably, patients in the >10-year postpartum group had the highest proportion of stage III disease (8.1%) compared with any other cohort. Whether this signal reflects true biological risk or is a product of limited statistical power requires validation in larger cohorts.

In conclusion, our study provides evidence that risk is elevated in HR+ breast cancers diagnosed in the immediate postpartum period, suggesting that the years shortly following childbirth may represent a distinct window of biological vulnerability. These findings highlight the importance of integrating reproductive history and gene expression profiling in the care of young women with breast cancer. They also underscore the need for larger, multi-institutional studies to validate and extend these observations, as well as for future clinical trials to systematically collect data on time since last childbirth to better understand the impact of postpartum breast cancer on tumor biology and treatment outcomes.

## Methods

### Study population

This retrospective study included women with HR+, HER2-negative early-stage breast cancer defined as stage I to III (AJCC anatomic staging) treated at a single academic center between January 1, 2011, and December 31, 2024. This study was conducted in accordance with the Declaration of Helsinki and approved by the University of California, Los Angeles Institutional Review Board (UCLA IRB, approval number 23-0376). As this was a retrospective medical record review study involving no direct patient contact or intervention, the UCLA IRB waived the requirement for informed consent. Eligible women were 45 years or younger at the time of Recurrence Score result collection and had complete parity data available. The recurrence score result was ordered at the treating physician’s discretion. Parity data were obtained through retrospective review of clinical notes and history and physical documentation in the electronic medical record. A total of 387 women were included in this analysis. Reporting adhered to the Strengthening the Reporting of Observational Studies in Epidemiology (STROBE) guidelines.

Individuals were categorized into cohorts based on date of childbirth prior to breast cancer diagnosis. Nulliparous patients were defined as those who had never had a full-term pregnancy prior to breast cancer diagnosis. The date of breast cancer diagnosis was defined as the date of the positive diagnostic biopsy. Patients who were pregnant at the time of breast cancer diagnosis were excluded from the analysis (*n* = 2). Initially, parous patients were subcategorized into three postpartum groups: 0–≤5 years, >5–≤ 10 years, and >10 years, respectively. Subsequently, patients were stratified by the exact number of years since their last childbirth. For patients with an unknown date of last childbirth but a known year (144 out of 232 parous patients), July 1 of that year was used as a proxy. For patients with an unknown day, the first day of the month was used. Postpartum duration was calculated by rounding to the nearest year.

Local recurrences were defined as disease occurring in the ipsilateral breast or draining lymph nodes following a breast cancer diagnosis, confirmed by radiologic imaging, physical examination, and/or pathological evaluation. Second primary breast malignancies were defined as a new histologically confirmed breast cancer diagnosis in the contralateral breast or in a new ipsilateral primary site distinct from the index tumor, occurring after completion of primary surgical treatment. Distant recurrences were defined as metastases to organs outside of the ipsilateral breast and draining lymph nodes after the primary breast cancer diagnosis, also confirmed by radiologic imaging, physical examination, and/or pathological evaluation. Patients without evidence of distant recurrence at the end of follow-up were censored at the date of last contact. Local recurrences, distant recurrences, second primary breast malignancies, and death from any cause were combined to calculate 4-year invasive disease-free survival (IDFS).

For tumor grade analysis, participants with available tissue and known tumor grade were included (*n* = 385). For Ki-67 analysis, participants with available tissue were included (*n* = 385), of whom 381 had evaluable results.

### Recurrence score, ER gene score, PR gene score, HER2 gene score

The Recurrence Score result, estrogen receptor (ER) gene score, progesterone receptor (PR) gene score, and HER2 gene score were obtained from Exact Sciences. The Recurrence Score result was determined using the 21-gene Oncotype DX^®^ test and reported on a continuous scale from 0 to 100. In addition to the Recurrence Score result, the Oncotype DX^®^ test report provides quantitative single-gene scores for ER, PR, and HER2. These single-gene scores represent the mRNA expression levels of the respective genes. For ER, PR, and HER2, the single-gene scores supplement receptor status determined by immunohistochemistry (IHC) but do so on a continuous numeric scale.

### Statistical analysis

Intergroup differences were assessed using *χ*² tests, Fisher’s exact tests, one-way analysis of variance (ANOVA), Kruskal–Wallis tests, Wilcoxon rank sum tests, or two-way ANOVA, as appropriate. Descriptive statistics characterized cohort distributions and assessed prevalence differences. Linear regression was applied to compare mean Recurrence Score result and gene expression scores for ER, PR, and HER2. Ordinal logistic regression evaluated the odds of higher Recurrence Score categories (0–15 low, 16–25 intermediate, and >25 high risk) and higher pathologic tumor grade following the Brant test for proportional odds. For models in which the recurrence score result was the outcome, adjustment was made for age at diagnosis and nodal status. Age adjustment was included because younger patients have been shown to present with more biologically aggressive disease. Nodal status adjustment was retained because the Oncotype DX^®^ test is not routinely recommended for lymph node-positive patients; without adjustment, differential testing rates across nodal categories could introduce selection bias. For models evaluating IDFS, the adjustment strategy was expanded to include condensed pTN stage (stage 1 vs. stage 2+), age at diagnosis, and year of breast cancer diagnosis (2015–2020 vs. 2021–2024). Stage II and III were combined given the limited number of stage III patients. Year of diagnosis was included to account for practice evolution during the study period, including publication of the RxPONDER trial results and FDA approval of CDK4/6 inhibitors for adjuvant use in high-risk HR-positive early breast cancer, both occurring in 2021. With a cohort size of 385, we had 80.3% power to detect a 4.5-point difference in mean Recurrence Score result between PPBC and non-PPBC patients. Power curves for the selected cohort sizes can be seen in Supplemental Fig. 1.

Kaplan-Meier analysis was additionally performed to assess 4-year IDFS, defined as the time from breast cancer diagnosis to first occurrence of local recurrence, distant recurrence, second primary breast malignancy, or death from any cause. Multivariable Cox proportional hazards regression was used to identify factors associated with IDFS. Hazard ratios (HRs) and 95% CIs were estimated. Overall models accounted for pathologic stage (1, 2+), patient age, and year of diagnosis (2015–2020, 2021–2024). A sensitivity analysis was additionally performed using recurrence-free survival (RFS), defined as time from breast cancer diagnosis to first occurrence of local recurrence, distant recurrence, or death, excluding second primary breast malignancies; results are reported in Supplementary Table 1 and Supplementary Fig. 5.

All statistical analyses were performed in R (version 4.4.1), with analyses conducted between July and November of 2025. A two-sided *p*-value < 0.05 was considered statistically significant with no multiple testing correction applied.

## Supplementary information

Supplementary Figures and Table 05282026.

## Data Availability

The datasets generated and/or analyzed during the current study are not publicly available due to patient privacy restrictions but are available from the corresponding author on reasonable request. The statistical analyses were performed using R (version 4.4.1). No custom code or software was developed for this study. Standard R packages were used as described in the Methods section. The analysis code is available from the corresponding author on reasonable request.

## References

[1] Londero, A. P., Bertozzi, S., Xholli, A., Cedolini, C. & Cagnacci, A. Breast cancer and the steadily increasing maternal age: are they colliding?*BMC Womens Health* **24**, 286 (2024). 10.1186/s12905-024-03138-4 PMC11092140 38745181

[2] Soerjomataram, I., Pukkala, E., Brenner, H. & Coebergh, J. W. W. On the avoidability of breast cancer in industrialized societies: older mean age at first birth as an indicator of excess breast cancer risk. *Breast Cancer Res. Treat.* **111**, 297 (2007). 10.1007/s10549-007-9778-2 PMC2491409 17932743

[3] Schedin, P. Pregnancy-associated breast cancer and metastasis. *Nat. Rev. Cancer* **6**, 281–291 (2006). 10.1038/nrc1839 16557280

[4] Klonoff-Cohen, H., An, R., Fries, T., Le, J. & Matt, G. E. Timing of breast cancer surgery, menstrual phase, and prognosis: Systematic review and meta-analysis. *Crit. Rev. Oncol. Hematol.* **102**, 1–14 (2016). 10.1016/j.critrevonc.2016.02.004 27066938

[5] Azim, H. A. et al. Prognosis of pregnancy-associated breast cancer: a meta-analysis of 30 studies. *Cancer Treat. Rev.* **38**, 834–842 (2012). 10.1016/j.ctrv.2012.06.004 22785217

[6] Hartman, E. K. & Eslick, G. D. The prognosis of women diagnosed with breast cancer before, during and after pregnancy: a meta-analysis. *Breast Cancer Res. Treat.* **160**, 347–360 (2016). 10.1007/s10549-016-3989-3 27683280

[7] Bakhuis, C. F. J. et al. Worse prognosis for breast cancer diagnosed in advanced pregnancy and shortly postpartum: an update of the Dutch pregnancy-associated breast cancer cohort. *Breast Cancer Res. Treat.* **214**, 191–204 (2025). 10.1007/s10549-025-07806-3 PMC12464139 40796679

[8] Amant, F. et al. The definition of pregnancy-associated breast cancer is outdated and should no longer be used. *Lancet Oncol.* **22**, 753–754 (2021). 10.1016/S1470-2045(21)00183-2 PMC8868503 34087122

[9] Amant, F. et al. Prognosis of women with primary breast cancer diagnosed during pregnancy: results from an international collaborative study. *J. Clin. Oncol.* **31**, 2532–2539 (2013). 10.1200/JCO.2012.45.6335 23610117

[10] Zhang, Z. et al. Young-onset breast cancer outcomes by time since recent childbirth in Utah. *JAMA Netw. Open* **5**, e2236763–e2236763 (2022). 10.1001/jamanetworkopen.2022.36763 PMC9568799 36239933

[11] Vohra, S. N. et al. Molecular and clinical characterization of postpartum-associated breast cancer in the carolina breast cancer study phase I-III, 1993-2013. *Cancer Epidemiol. Biomark. Prev.* **31**, 561–568 (2022). 10.1158/1055-9965.EPI-21-0940 PMC8901538 34810211

[12] Goddard, E. T. et al. Association between postpartum breast cancer diagnosis and metastasis and the clinical features underlying risk. *JAMA Netw. Open* **2**, e186997–e186997 (2019). 10.1001/jamanetworkopen.2018.6997 PMC6484560 30646210

[13] Shagisultanova, E. et al. Overall survival is the lowest among young women with postpartum breast cancer. *Eur. J. Cancer* **168**, 119–127 (2022). 10.1016/j.ejca.2022.03.014 PMC9233962 35525161

[14] Peña-Enríquez, R. et al. Molecular characterization of pregnancy-associated breast cancer and insights on timing from GEICAM-EMBARCAM study. *npj Breast Cancer* **11**, 1–16 (2025). 10.1038/s41523-025-00718-x PMC11807221 39922815

[15] Jindal, S. et al. Postpartum breast cancer has a distinct molecular profile that predicts poor outcomes. *Nat. Commun. 2021* **12**, 1–15 (2021). 10.1038/s41467-021-26505-3 PMC8566602 34732713

[16] Borges, V. F. et al. 32P Genomic risk classification and whole transcriptome analysis of HR+/HER2- post-partum breast cancers: A FLEX sub study. *ESMO Open* **9**, 103040 (2024).

[17] Brufsky, A. M. et al. MammaPrint predicts chemotherapy benefit in HR+HER2- early breast cancer: FLEX Registry real-world data. *JNCI Cancer Spectr.* **9**, pkaf079 (2025). 10.1093/jncics/pkaf079 PMC12413225 40796181

[18] Sparano, J. A. et al. Prospective validation of a 21-gene expression assay in breast cancer. *N. Engl. J. Med.* **373**, 2005–2014 (2015). 10.1056/NEJMoa1510764 PMC4701034 26412349

[19] Klebaner, D. et al. Impact of timing and breastfeeding on postpartum breast cancer diagnostic patterns and outcomes. *Breast Cancer Res Treat.* **214**, 79–86 (2025). 10.1007/s10549-025-07796-2 40739382

[20] Lyons, T. R., Schedin, P. J. & Borges, V. F. Pregnancy and breast cancer: When they collide. *J. Mammary Gland Biol. Neoplasia* **14**, 87–98 (2009). 10.1007/s10911-009-9119-7 PMC2693784 19381788

[21] Virassamy, B. et al. Parity and lactation induce T cell mediated breast cancer protection. *Nature* **649**, 1–3 (2025). 10.1038/s41586-025-09713-5 PMC12779547 41115453

[22] Sparano, J. A. et al. Adjuvant Chemotherapy Guided by a 21-Gene Expression Assay in Breast Cancer. *N. Engl. J. Med.* **379**, 111–121 (2018). 10.1056/NEJMoa1804710 PMC6172658 29860917

[23] Kalinsky, K. et al. 21-Gene assay to inform chemotherapy benefit in node-positive breast cancer. *N. Engl. J. Med.* **385**, 2336–2347 (2021). 10.1056/NEJMoa2108873 PMC9096864 34914339

[24] Mamounas, E. P. et al. Association between the 21-gene recurrence score assay and risk of locoregional recurrence in node-negative, estrogen receptor-positive breast cancer: results from NSABP B-14 and NSABP B-20. *J. Clin. Oncol.* **28**, 1677–1683 (2010). 10.1200/JCO.2009.23.7610 PMC2849763 20065188

[25] Cicenas, J. et al. CDK inhibitors and FDA: approved and orphaned. *Cancers* **16**, 1555 (2024). 10.3390/cancers16081555 PMC11049492 38672637

