# Supplementary material for: Identifying the window of aggressive postpartum breast cancer based on the 21-gene Oncotype DX® test in women with HR+, HER2-negative breast cancer
Source: NPJ Breast Cancer. 2026 Jul 1;12:139. doi: 10.1038/s41523-026-01002-2 (PMC13686956; doi:10.1038/s41523-026-01002-2)
Supplement: Supplementary file 1 [file 41523_2026_1002_MOESM1_ESM.pdf]

Supplementary Figure 1: Power curves for difference in recurrence score by cohort size

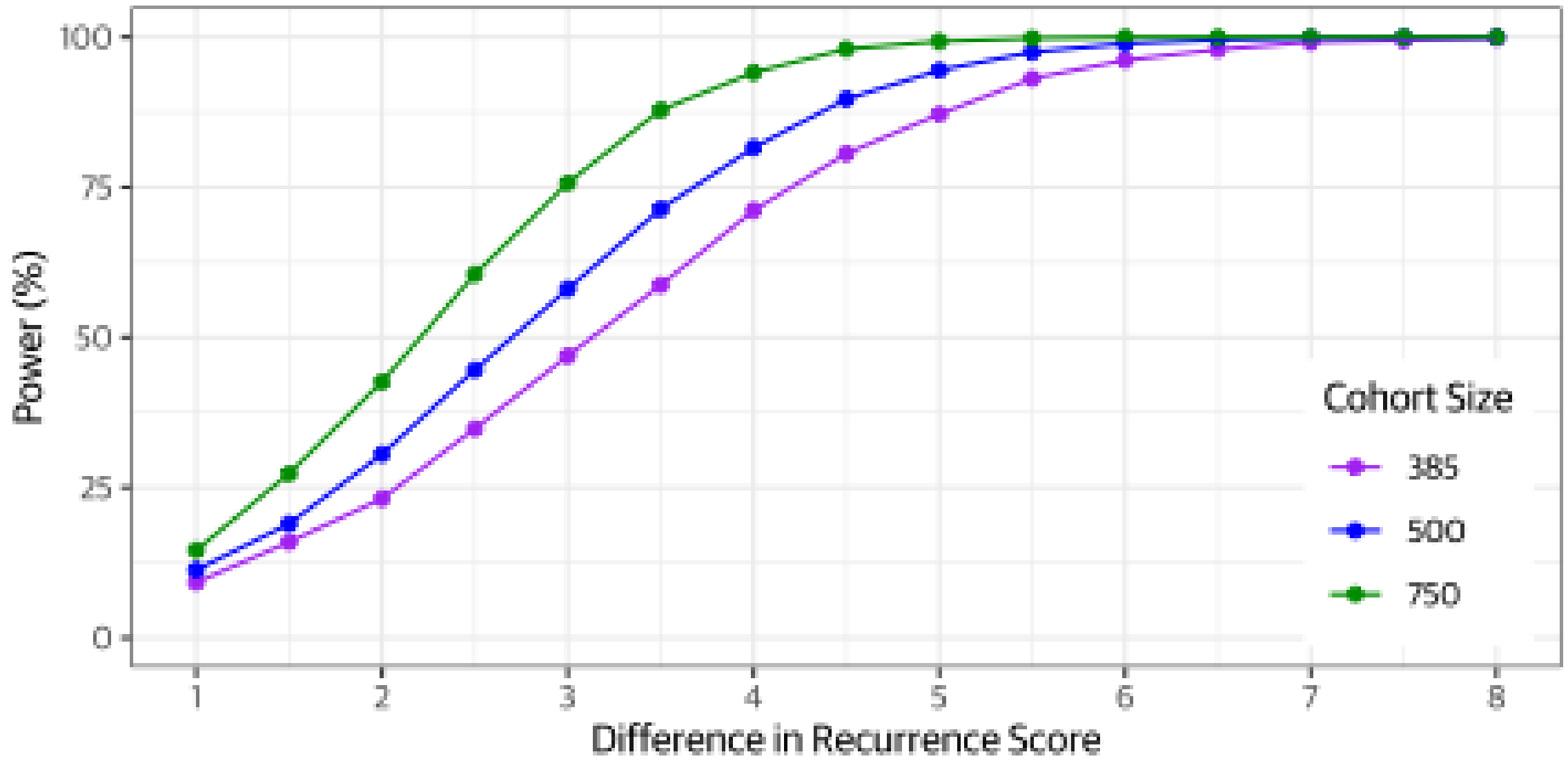

Supplemental Figure 1: Line plot for power to detect a difference in recurrence score between PPBC and non-PPBC cases based on cohort size. The purple line represents the available cohort used for analysis (n = 385). 10,000 trials of the appropriate sample size assigned patients to PPBC or non-PPBC at a proportion of 12.2%. Recurrence Score was generated from a normal distribution with mean 24.4 (PPBC) or 24.4 minus the difference (non-PPBC) with a standard deviation of 10.

Supplemental Figure 2: ER gene score by postpartum year

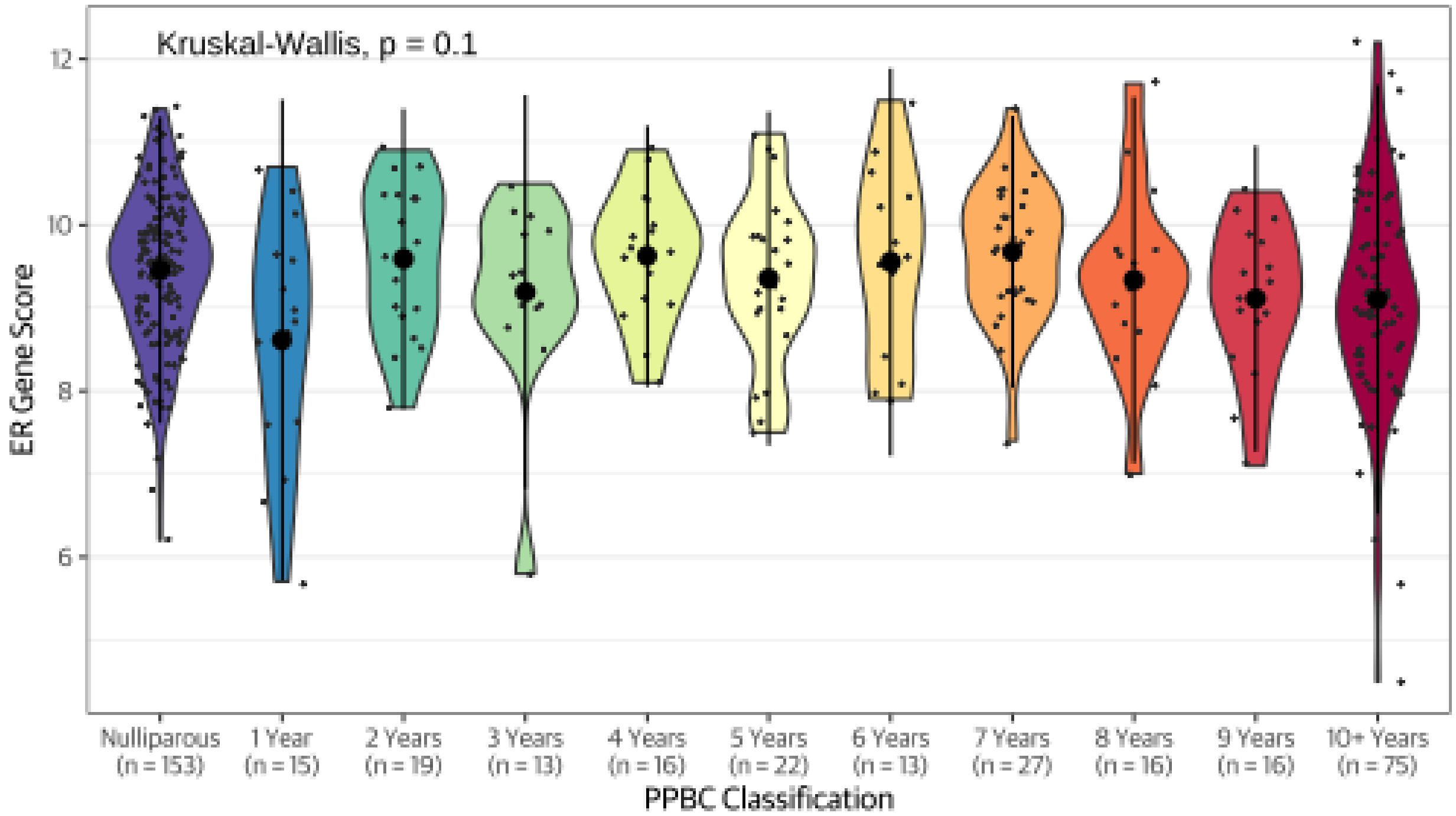

Supplemental Figure 2: Violin plot for estrogen receptor gene score by PPBC classification. Each dot represents values for one patient. Kruskal-Wallis was used to test for global differences.

Supplemental Figure 3: PR gene score by postpartum year

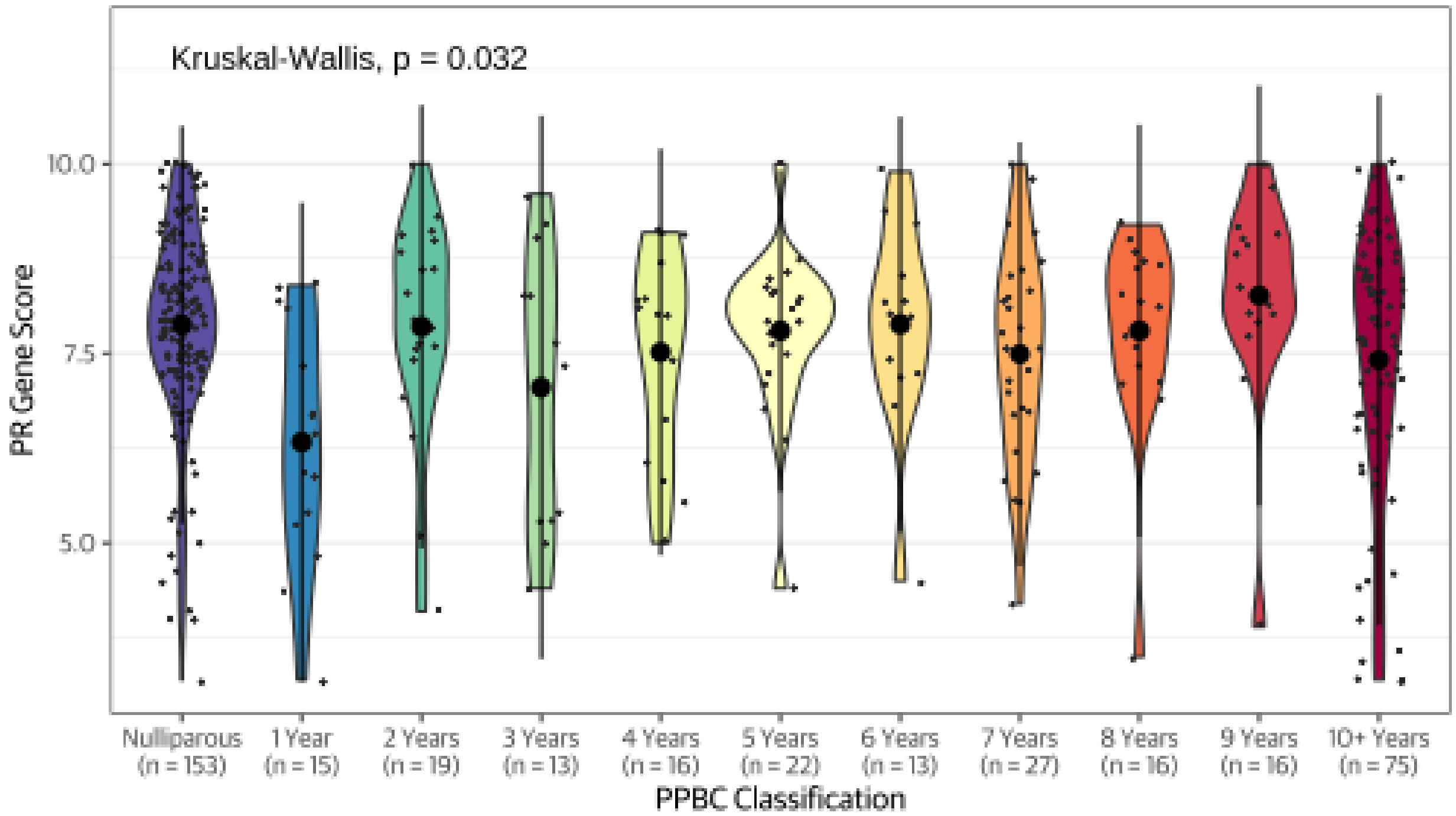

Supplemental Figure 3: Violin plot for progesterone receptor gene score by PPBC classification. Each dot represents values for one patient. Kruskal-Wallis was used to test for global differences.

Supplemental Figure 4: Recurrence Score and age at first childbirth, age of menarche

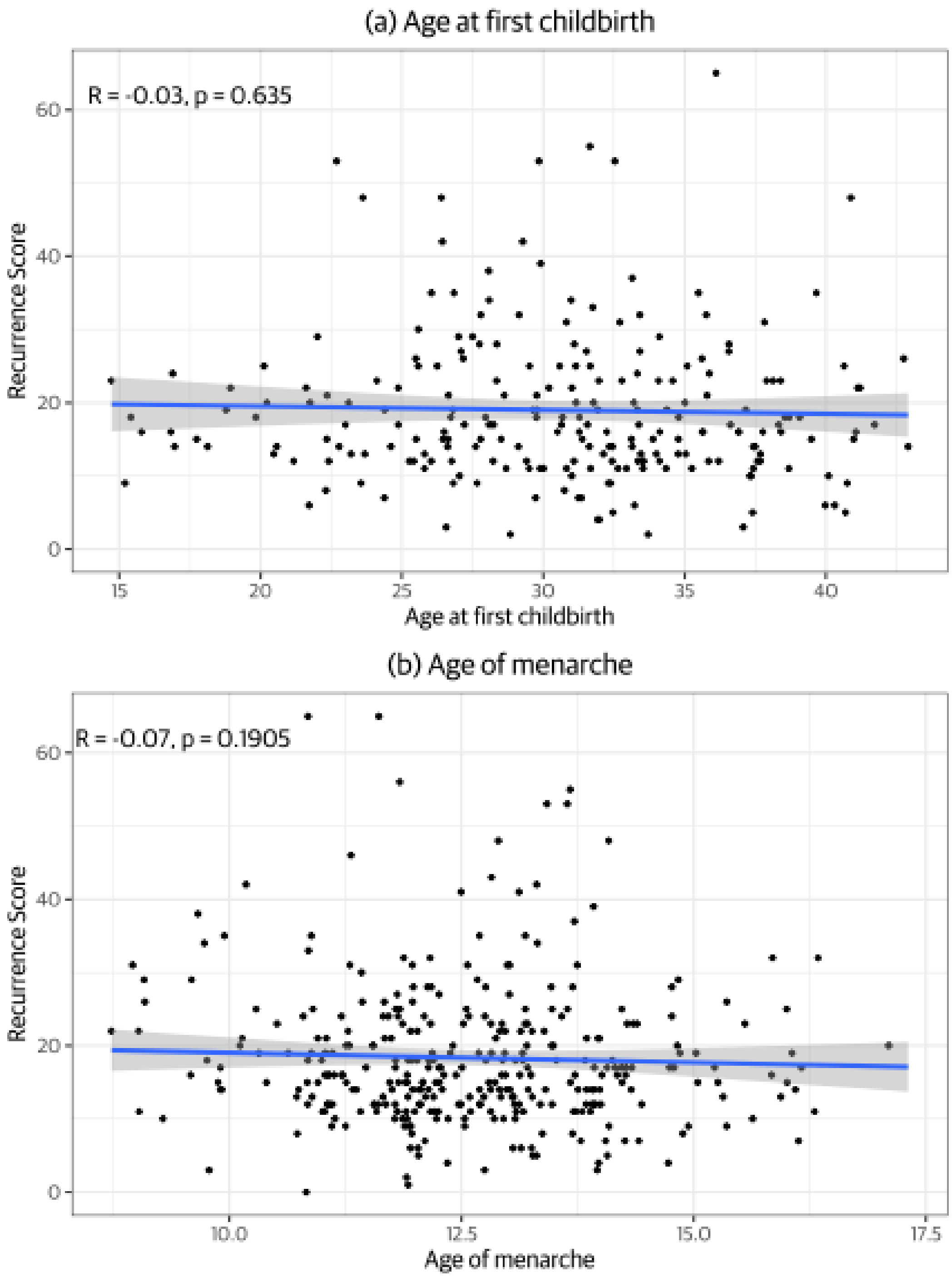

Supplemental Figure 4: Scatter plots for recurrence score vs age at first childbirth (a) and age of menarche (b). Each dot represents one patient. R is Pearson's correlation coefficient, and p-value is from the two-sided t-test. Random noise added to age at menarche in plot (b) for readability.

Supplemental Figure 5: Recurrence-free survival by cohort

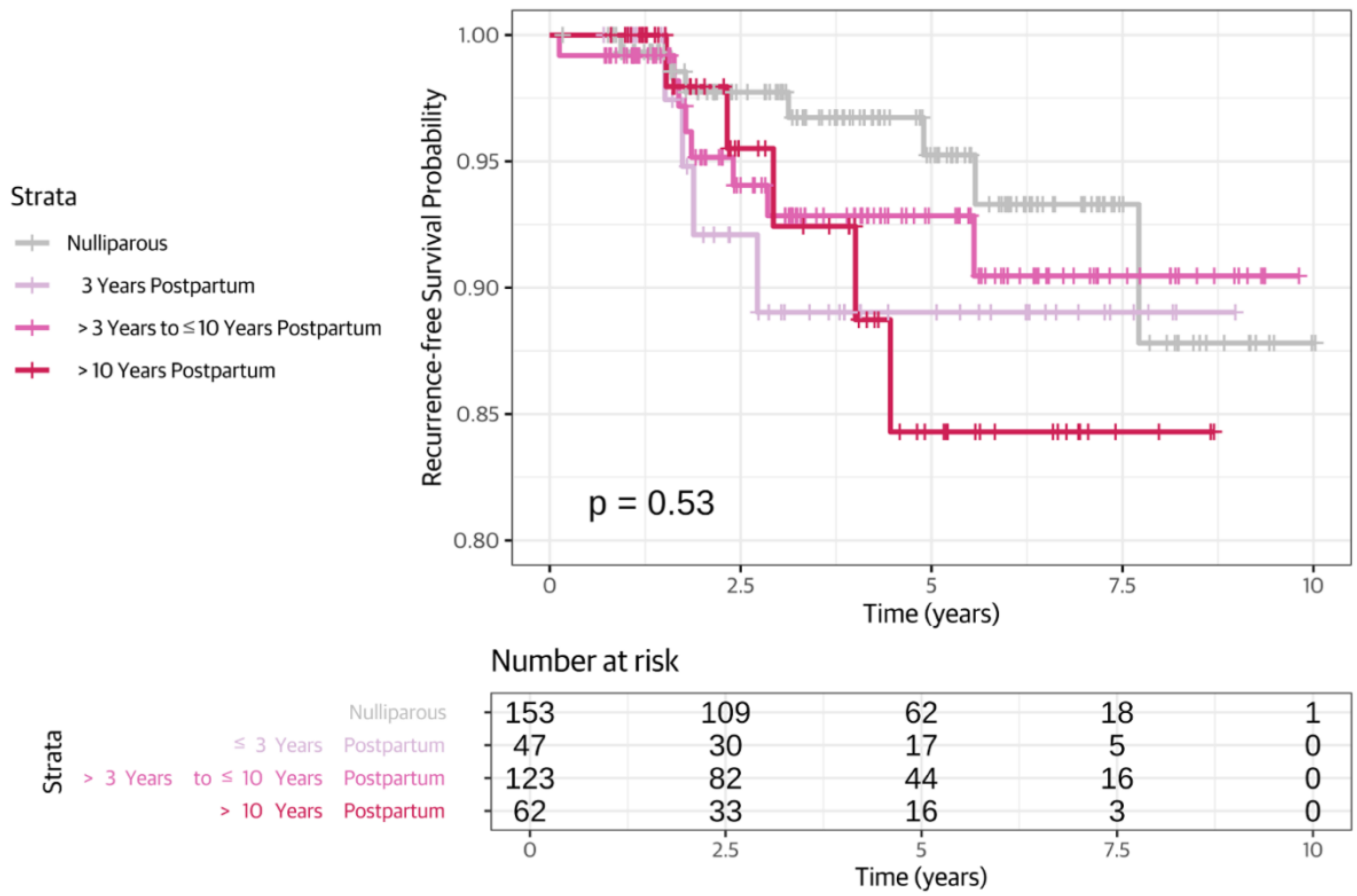

Supplemental Figure 5: Kaplan-Meier curve and risk table for invasive disease-free survival by PPBC classification. Patients were censored (vertical bars) at last follow-up. P-value calculated using a log-rank test for differences between groups.

Supplemental Table 1: Comparison of effect of different definitions of postpartum cohort on recurrence-free survival

	Recurrence-Free Survival	
**PPBC Definition**	**HR (95% CI)**	**p-value**
**PPBC, ≤ 5 years pp**		
≤ 5 years pp	2.60 (0.89, 7.61)	0.081
> 5 to ≤ 10 years pp	2.87 (0.80, 10.22)	0.10
> 10 years pp	5.32 (1.40, 20.24)	0.014*
**PPBC, ≤ 3 years pp**		
≤ 3 years pp	2.34 (0.68, 8.06)	0.18
> 3 to ≤ 10 years pp	3.01 (0.96, 9.44)	0.059
> 10 years pp	5.48 (1.42, 21.17)	0.014*

Supplemental Table 1: Models adjusted for pTN stage (1, 2+) and age at diagnosis, and the invasive disease-free survival model additionally adjusted for year of breast cancer diagnosis (2015 – 2020, 2021 – 2024); Reference group is nulliparous; Increasing number of * represent statistical significance at alpha = 0.05, 0.01, and 0.001; PPBC = postpartum breast cancer; pp= postpartum; CI = confidence interval; HR = hazards ratio
